# Optimization of Extraction Method of Anthocyanins from Red Cabbage

**DOI:** 10.3390/molecules28083549

**Published:** 2023-04-18

**Authors:** Auryclennedy Calou de Araújo, Josivanda Palmeira Gomes, Francilânia Batista da Silva, Jarderlany Sousa Nunes, Francislaine Suelia dos Santos, Wilton Pereira da Silva, João Paulo de Lima Ferreira, Alexandre José de Melo Queiroz, Rossana Maria Feitosa de Figueirêdo, Geovani Soares de Lima, Lauriane Almeida dos Anjos Soares, Ana Paula Trindade Rocha, Antonio Gilson Barbosa de Lima

**Affiliations:** 1 Center for Technology and Natural Resources, Academic Unit of Agricultural Engineering, Federal University of Campina Grande, Campina Grande 58429-900, Brazil; 2 Agroindustry Coordination, Federal Institute of Sertão Pernambucano, Ouricuri 56200-000, Brazil; 3 Agrifood Science and Technology Center, Academic Unit of Agricultural Sciences, Federal University of Campina Grande, Pombal 58840-000, Brazil

**Keywords:** *Brassica oleracea*, pigments, natural dyes, bioactive compounds

## Abstract

Among the vegetables that stand out for their high concentration of anthocyanins, red cabbage appears as one of the most-used sources of these pigments in food production and it is considered a suitable raw material for the extraction of natural dye. Therefore, the objective was to carry out the production of natural extracts from red cabbage, under different conditions, varying the solvent, type of pre-treatment, pH range, and processing temperature during the concentration of the extracts. The anthocyanins were extracted from red cabbage using the following solvents: distilled water, 25% ethyl alcohol, and 70% ethyl alcohol. The raw material was divided into two groups, the first was subjected to a drying pre-treatment at 70 °C for 1 h and for the second group, the extraction was performed with the raw material in natura. Two pH ranges of 4.0 and 6.0 and extraction temperatures of 25 °C and 75 °C were used in the extracts, resulting in 24 formulations. The extracts obtained were analyzed for colorimetric parameters and anthocyanins. The results of anthocyanins show that the methodology that uses 25% alcohol, pH 4.0, and processing temperature of 25 °C produces a reddish extract and better results in the extraction, presenting average values of 191.37 mg/100 g of anthocyanins, being 74% higher compared to the highest values obtained in the other extracts where the same raw material was used and the solvents differed.

## 1. Introduction

In the food industry, dyes are the additives used to check, intensify, or standardize the color, which can restore the color lost during processing and storage [1]. The intent to maintain or add color to food can be considered one of the main applications of dyes, whether natural or artificial. Although the food ingredients industry is more committed to the development of synthetic dyes, as they have greater stability, attractive color, and their production is low cost, natural food dyes are gradually gaining ground in this segment due to the change in the lifestyle of consumers, adoption of healthy eating, and increased concerns about possible adverse health effects and environmental damage caused by artificial colors [2].

As an example, consumption of some artificial dyes is associated with an increase in hyperactive behavior in children. Studies that report cases of allergic reactions by susceptible individuals who consume artificially colored foods are also common. In addition, the preference for the application of natural dyes in food products can provide technological resources and bioactive functionalities, resulting in a final product with greater added value [3]. In this sense, the processing of natural raw materials, still little explored, should be considered by the food industry for the development of new products such as dyes, as it may attract new customers according to the needs of consumers [4]. 

In the USA and the European Union, different natural dyes have been extracted and exploited commercially, and anthocyanins are among them [5]. Despite the large number of natural dyes intended for application in the food industry, anthocyanins, carotenoids, phycobiliproteins, betalains, and chlorophylls stand out, being the most common pigments for applications in food [6].

Anthocyanins are flavonoids and can appear in colors ranging from red to blue, depending on the pH at which they are found; these pigments are recognized for several bioactive properties such as antioxidant, anti-inflammatory, hypoglycemic, and chemopreventive effects [7]. Similar to other natural pigments, the anthocyanins are unstable and could decompose during the extraction, purification, production, and storage. The factors that affect the potent of anthocyanin could include the chemical structure of color, pH, and temperature [8]. However, the endurance of anthocyanin depends on many other factors such as ascorbic acid, metals, sugar, oxygen, light, and enzymes in the manufacturing process and polymerization [9].

According to studies on raw materials with high potential for pigmentation, red cabbage is one of the vegetables that make up the group of vegetables rich in anthocyanins. For this and other characteristics, such as the low commercial value and the ease of extracting pigments, the anthocyanins of this vegetable still present another differential in relation to the others, which is the greater stability due to its acidic environment. Another important feature is that their application is not restricted to acidic foods, and can also color neutral foods. Currently, its applicability has focused on coloring drinks, sweets, dry mixed concentrates, chewing gum, yogurts, and sauces [10]. To obtain natural dyes, it is necessary to carry out an extraction process for these compounds. However, some natural bioactives that are present in these dyes can be degraded due to the applied processing conditions [11].

From a scientific point of view, considering red cabbage as a promising source for the extraction of natural pigments, as it is a vegetable rich in anthocyanins, of low cost and wide availability, the present study aims to define favorable conditions for the extraction of anthocyanins using solvents of reduced high value, thus, producing natural extracts of red cabbage, under different conditions, varying the type of solvent, type of pre-treatment of the raw material, pH range, and processing temperature.

## 2. Results and Discussion

In Table 1 the quantitative results of anthocyanins present in the extracts obtained from different solvents (water, 25 and 70% alcohol) and under different extraction conditions are presented, showing the variations in the treatment of the raw material before extraction (in natura and with pre-drying), the pH range of the solution (4.0 and 6.0), and the extraction processing temperature (25 and 75 °C).

It is observed that all the results present minimum significant deviation and a coefficient of variation lower than 0.35. The extracts 02 and 08 do not differ from each other as both use water as the extractor solvent and raw material with pre-drying in an oven at 70 °C for 1 h. The variation in these formulations occurs only by pH and processing temperature.

All results obtained in this study are higher than the anthocyanin value reported by Vergara et al. [12], who quantified 9.32 mg/100 g of anthocyanins in formulation from blackberry, *butia,* and red *pitanga* pulps. In the studies, the authors proposed the study of bioactives in a mix of pulps used to formulate chewable candies. The anthocyanin content presented here also exceeds those obtained by Barbosa et al. [13], in which the authors report 4.0 mg/100 g of anthocyanins in an aqueous extract obtained from diluted powdered hibiscus.

The values for extracts 09, 11, and 15 (96.47, 191.37, and 171.34 mg/100 g, respectively), with 25% alcohol in the formulation, are close to the value quantified by Huaranca-Huarchaya et al. [14], who obtain 147.99 mg/100 g of anthocyanins in an acidified alcoholic extract obtained from Alaybili (Vaccinium floribundum kunth) when they study the kinetics of thermal degradation of anthocyanins. These values are also close to the result obtained by Moura et al. [15], who quantify 138.96 mg/100 g of anthocyanins in a 50% hydroalcoholic extract obtained from hibiscus calyx when characterizing and quantifying bioactive compounds in the plant.

The averages of the other extracts are similar to the results obtained by Silva et al. [16], who report 20.60 mg and 62.57 mg/100 g of anthocyanins for hydroalcoholic extracts (80%) obtained from the dried and ground pomace of malbec and petit verdot grape varieties, respectively. Wang et al. [17], when studying extraction of total anthocyanins in purple sweet potato through ultrasound-assisted enzymatic extraction (UAEE), obtain the best results of 2.27 mg/g in 78% ethanol concentration, material-liquid ratio of 1:15 g/mL, and pH 4.5.

According to the arrangement of the data in Table 1, it is observed that there is no standardization from the highest to the lowest value or the opposite, in relation to the statistical difference. However, it is noticeable that the highest values of anthocyanins in each table are always associated with the extract with pH 4.0 and temperature of 25 °C. This fact corroborates what was described by Barbosa et al. [18], where the authors mention that the highest results of anthocyanins are generally associated with lower pH numbers, since phenolic compounds have greater affinity with acidified media/environments. Temperature is one of the factors that most influence the stability of anthocyanins, more specifically, monomeric anthocyanins and the intensity of the corresponding dye tend to decrease with the time-temperature combination [19]. 

Table 2 shows the values of the colorimetric parameters for the extracts that use water, 25% alcohol, and 70% alcohol as extracting solvent.

It is noticed that the luminosity (L*) is directly influenced by the pH and the extraction temperature in all the solvents used. The lowest values of L* are found when we use 25% alcohol as solvent, indicating that less translucent extracts are obtained when this solvent is used at this concentration. This phenomenon can be explained when we look at the table with the values of anthocyanins present, where the extracts that are have 25% alcohol applied have higher values than the others, showing greater pigmentation, that is, a more efficient extraction. Rigolon and Stringheta et al. [20], when analyzing *juçara* fruits, found a luminosity of 24.32 ± 0.08, a value close to that presented in the extraction method with 25% alcohol and lower pH (4.0), using a temperature of 25 °C.

The parameter a* demonstrates great variation both within the type of solvent and also in the variables studied, where the maximum value shown is 24.433 and the minimum is 0.3566. This parameter is responsible for indicating the presence of the red color in the extracts, so the greater the value of a*, the greater the influence of red on the color of the extracts, while the b* parameter indicates the incidence of yellow/blue in the sample, as Barretto et al. [21] state. Thuy et al. [22], when studying the colorimetric parameters of blackberry pulp powders (*Morus* sp.) obtained at temperatures of 60 and 70 °C, found lower values for a* between 4.55 to 6.00 and higher values for L* of 59.93 and 64.13, respectively. The chroma corresponds to the color saturation of the samples, that is, their intensity. Values close to zero correspond to neutral colors (gray tones), while values close to 60 indicate bright colors, being considered a quantitative attribute of the color and used to determine the degree of difference of a tone in comparison with a gray color with the same luminosity. The higher the chroma values, the greater the color intensity of the samples perceived by humans [23]. Therefore, the chromaticity results in values ranging from 7.917 to 22.566, which corresponds to low saturation, indicating neutral colors, less bright to human perception. It is also noticed that the a* parameter has a greater influence on the color saturation of the extracts, a result also found by Madalão [24] for the analysis of *juçara* pulp.

The hue angle, according to the CIELab system, translates the tone and can vary from 0° to 360°, where at 0° there is a predominance of the red color, and at 90° the predominant tone is yellow. At 180°, there is a predominance of the green tone and at 270° the predominant blue tone is perceived [25]. Thus, we can observe that, according to the results found in this study, the extracts do not show a defined behavior, but remain in the first quadrant of the colored solid, with a predominant shade of red.

## 3. Materials and Methods

### 3.1. Obtaining and Processing Raw Materials

The red cabbages were purchased at a local market and transported to the Laboratory for the Storage and Processing of Agricultural Products (LAPPA) at the Federal University of Campina Grande (UFCG), PB, Brazil, where they were received and selected according to size and weight.

In the preparation of the raw material, the cabbages were defoliated, the external and damaged leaves were discarded, and the central stalks were removed and sanitized (200 ppm of active chlorine/10 min).

### 3.2. Obtaining Red Cabbage Extracts

To obtain the extracts, three types of solvents were used: water, 25% ethyl alcohol, and 70% ethyl alcohol. In the preparation of the raw material, part of the leaves were submitted to a pre-treatment before contact with the extracting solvent, performing a pre-drying in an air circulation oven (Fanem, model 320, Guarulhos, São Paulo, Brazil) at a temperature of 70 °C for 1 h. From this procedure, two raw materials for extraction were obtained: the fresh leaves of red cabbage in natura and the leaves that received pre-drying.

**Figure 1:**
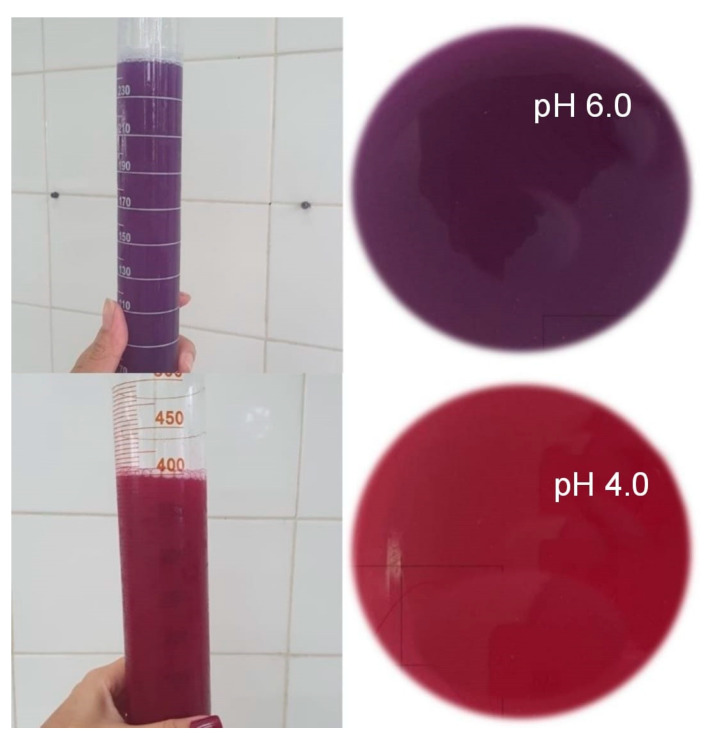
Red cabbage extracts with pH 6.0 and 4.0.

To obtain the extract, the methodology described by Nazaré et al. [26] was used, in the proportion 1:2 *w*/*v*, in which for every 50 g of raw material, 100 mL of extracting solvent was added. After homogenization of the leaves with the solvent in a blender (Arno, model Power max 1000 W, Itapevi, São Paulo, Brazil) for 5 min, the extracts had the pH adjusted with hydrochloric acid (HCl) 1.0 N until reaching the range of pH 4.0 and with sodium hydroxide (NaOH) 1.0 N until reaching the pH 6.0 range (Figure 1). In this pH adjustment step, the extracts underwent changes in color.

After pH adjustments, the extracts were submitted to different temperatures and kept under these conditions for 30 min, applying two temperatures, 25 and 75 °C. Samples subjected to a temperature of 75 °C were heated in a water bath (SPLabbor, Model SP-06/100ED, São Paulo, Brazil). The mixtures were kept at rest for 24 h at a refrigerated temperature of 10 °C (±2), in glass containers with lids and wrapped in aluminum foil, being homogenized by manual and slow stirring every 6 h.

**Figure 2:**
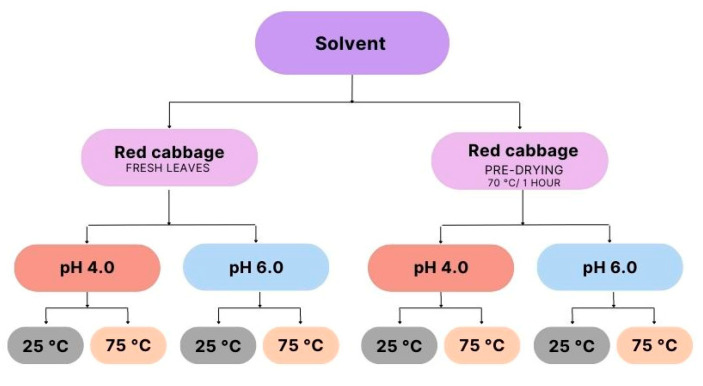
Steps for obtaining red cabbage extracts.

After resting, the extracts were subjected to two filtrations, the first in a stainless steel sieve and the second in a vacuum, with the aid of a Büchner funnel. The samples were concentrated in a vacuum rotary evaporator (QUIMIS, Model Q-344B2, Diadema, São Paulo, Brazil) until the solvent was completely removed. Figure 2 describes the steps for obtaining red cabbage extracts. 

The experiment consisted of a total of 24 samples of extracts under different extraction conditions, with (eight) samples for each type of solvent used, two types of treatments for the raw material, two pH ranges, and two different temperatures.

For a better organization of the data, the extracts were coded with numbers from 1 to 24, where extracts 01 to 08 are the extracts where water was used as an extracting agent, extracts 09 to 16 refer to the extracts where 25% alcohol was used as extractor solvent, and extracts number 17 to 24 are the extracts with 70% alcohol in their formulation.

Table 3 shows the codification and specifications of the samples with their respective extraction conditions.

### 3.3. Characterization of Extracts

The extracts obtained were evaluated for total anthocyanin content and colorimetric parameters. The determination of anthocyanins was performed according to the method described by Francis [27], in which 0.2 g of the sample was dissolved in 10 mL of the extracting solution, ethanol–HCL (1.5 N) in the proportion 85:15, in a dark environment and left to rest for 24 h under refrigeration. Readings were performed in a spectrophotometer (Coleman, model 35-D, Santo André, São Paulo, Brazil) and the results expressed in mg tannic acid equivalent (TAE) at a wavelength defined as 535 nm for absorbance.

The color characteristics were evaluated using the Minolta colorimeter (Chroma meter CR400 model) by measuring the color parameters L* (brightness, which varies from black to white), a* (chromaticity that changes from green to red, where +a indicates red and −a indicates green), and b* (chromaticity from blue to yellow, where +b indicates yellow and –b indicates blue). The chroma C* (saturation index) and the hue angle h* (tonality angle) were determined by Equations (1) and (2), respectively:

(1) $$\mathrm{Chroma}=\sqrt{{(a}^{2}+b^{2})}$$

(2) $${}^{\circ}\mathrm{Hue}=\tan^{-1}\frac{b^{*}}{a^{*}}$$

where a* = parameter variation a*; b* = parameter variation b*.

### 3.4. Statistical Analysis

Statistical analysis of the parameters studied were carried out in plots divided into groups according to the types of solvents applied and the condition of the raw material, with the entire experiment being carried out in triplicate. 

The data obtained were submitted to analysis of variance (ANOVA), and mean comparison test according to Tukey’s test at 5% probability, using the statistical program Sisvar version 5.6 [28].

## 4. Conclusions

Alcohol 25% is a viable and efficient extractor solvent for obtaining anthocyanin extracts. Purple cabbage in its in natura state is a raw material suitable for extracting pigments. The lowest pH (4.0) and processing temperature of 25 °C are the most favorable conditions for the extraction and maintenance of anthocyanins in red cabbage extracts. The extracts obtained in this study show a predominance of reddish coloration.

With the results expressed in the present work, it can be concluded that the extract that presents the best result in relation to the quantification of anthocyanins is the alcoholic extract 25% in natura, since it is non-toxic and has a low cost. 

The present work is a very promising preliminary result that serves as a starting point for future projects, where the conditions of toxicity and microbiological safety of the extracts can be evaluated for the conduction of an applicability test in a food matrix. In addition, other ways of obtaining the final product can be researched, such as a powdered dye, an anthocyanin-enriched dyestuff, and others.

## Figures and Tables

**Table 1:** Anthocyanin content in red cabbage extracts obtained from different solvents (water, 25 and 70% alcohol) and under different extraction conditions.

Solvent: Water	Extracts ^1^							
**Anthocyanins** **(mg/100 g)**	**01**	**02**	**03**	**04**	**05**	**06**	**07**	**08**
	34.33 ± 0.02 ^e^	44.24 ± 0.10 ^b^	38.77 ± 0.02 ^c^	49.21 ± 0.02 ^a^	24.67 ± 0.07 ^g^	29.91 ± 0.02 ^f^	37.99 ± 0.13 ^d^	44.53 ± 0.05 ^b^
**Solvent:** Alcohol 25%	**Extracts ^2^**							
**Anthocyanins** **(mg/100 g)**	**09**	**10**	**11**	**12**	**13**	**14**	**15**	**16**
	96.47 ± 0.05 ^c^	45.68 ± 0.02 ^f^	191.37 ± 0.40 ^a^	48.35 ± 0.05 ^d^	35.52 ± 0.09 ^h^	44.35 ± 0.05 ^g^	171.34 ± 0.06 ^b^	46.92 ± 0.14 ^e^
**Solvent:** Alcohol 70%	**Extracts ^3^**							
**Anthocyanin** **(mg/100 g)**	**17**	**18**	**19**	**20**	**21**	**22**	**23**	**24**
	44.86 ± 0.05 ^d^	31.71 ± 0.04 ^g^	48.06 ± 0.02 ^b^	48.73 ± 0.04 ^a^	40.24 ± 0.02 ^e^	26.35 ± 0.02 ^h^	45.16 ± 0.05 ^c^	32.60 ± 0.11 ^f^

^1^ MSD = 0.367; GA = 37.95; CV = 0.34%; ^2^ MSD = 0.021; GA = 85.00; CV = 0.01%; ^3^ MSD = 0.037; GA = 39.72; CV = 0.03%. MSD—minimum significant deviation; GA—general average; CV—coefficient of variation; means followed by the same letters do not differ statically by Tukey’s test, at 5% probability.

**Table 2:** Values of luminosity (L*), red intensity (a*), yellow/blue intensity (b*), Chroma and hue angle in red cabbage extracts obtained from different solvents (water, 25 and 70% alcohol) and under different extraction conditions.

Solvent: Water	Colorimetric Parameters ^1^				
Extracts	L*	a*	b*	Chroma	°hue
01	20.880 ± 0.19 ^b^	20.400 ± 0.49 ^b^	7.660 ± 0.46 ^c^	21.90 ± 0.07 ^b^	20.579 ± 0.03 ^ab^
02	17.743 ± 0.11 ^d^	11.413 ± 0.02 ^e^	2.733 ± 0.04 ^e^	11.736 ± 0.07 ^e^	13.467 ± 0.11 ^c^
03	23.826 ± 0.18 ^a^	24.433 ± 0.17 ^a^	−2.46 ± 0.05 ^b^	24.557 ± 0.05 ^a^	5.765 ± 0.05 ^d^
04	19.296 ± 0.02 ^c^	16.370 ± 0.03 ^dc^	5.176 ± 0.06 ^d^	17.169 ± 0.07 ^c^	17.548 ± 0.04 ^bc^
05	19.543 ± 0.28 ^c^	15.136 ± 0.17 ^d^	1.010 ± 0.11 ^fe^	15.170 ± 0.11 ^d^	3.8147 ± 0.05 ^d^
06	19.406 ± 0.71 ^c^	1.826 ± 0.04 ^g^	0.856 ± 0.21 ^f^	2.024 ± 0.07 ^g^	24.999 ± 0.04 ^a^
07	20.586 ± 0.09 ^b^	17.623 ± 0.48 ^c^	−5.530 ± 0.16 ^a^	18.470 ± 0.07 ^c^	17.420 ± 0.05 ^bc^
08	17.370 ± 0.08 ^d^	8.356 ± 0.02 ^f^	1.940f ± 0.04 ^e^	8.578 ± 0.04 ^f^	13.069 ± 0.03 ^c^
**Solvent: Alcohol 25%**	**Colorimetric Parameters ^2^**				
**Extracts**	L*	a*	b*	**Chroma**	**°hue**
09	14.576 ± 0.04 ^h^	0.3566 ± 0.12 ^g^	1.993 ± 0.09 ^d^	2.028 ± 0.03 ^g^	79.763 ± 0.02 ^a^
10	20.610 ± 0.09 ^b^	18.910 ± 0.12 ^b^	4.060 ± 0.04 ^c^	19.341 ± 0.02 ^b^	12.118 ± 0.02 ^dc^
11	17.880 ± 0.06 ^f^	7.826 ± 0.05 ^d^	0.636 ± 0.04 ^e^	7.852 ± 0.11 ^d^	4.649 ± 0.03 ^e^
12	19.550 ± 0.01 ^c^	16.520 ± 0.04 ^c^	5.456 ± 0.02 ^b^	17.397 ± 0.02 ^c^	18.278 ± 0.11 ^b^
13	16.750 ± 0.06 ^g^	2.290 ± 0.03 ^f^	0.540 ± 0.03 ^e^	2.353 ± 0.04 ^f^	13.275 ± 0.03 ^c^
14	19.283 ± 0.03 ^d^	7.640 ± 0.07 ^d^	1.846 ± 0.03 ^d^	7.860 ± 0.02 ^d^	13.590 ± 0.04 ^c^
15	22.473 ± 0.04 ^a^	20.336 ± 0.05 ^a^	−3.013 ± 0.04 ^a^	20.558 ± 0.04 ^a^	8.428 ± 0.02 ^ed^
16	18.176 ± 0.06 ^e^	7.276 ± 0.13 ^e^	2.050 ± 0.01 ^d^	7.559 ± 0.03 ^e^	15.735 ± 0.02 ^cb^
**Solvent: Alcohol 70%**	**Colorimetric Parameters ^3^**				
**Extracts**	L*	a*	b*	**Chroma**	**°hue**
17	18.356 ± 0.02 ^c^	18.483 ± 0.07 ^c^	7.053 ± 0.02 ^a^	19.783 ± 0.07 ^c^	20.887 ± 0.02 ^a^
18	16.663 ± 0.01 ^h^	10.396 ± 0.09 ^g^	2.866 ± 0.07 ^d^	10.784 ± 0.01 ^g^	15.417 ± 0.07 ^c^
19	19.783 ± 0.02 ^b^	21.713 ± 0.20 ^a^	6.146 ± 0.06 ^b^	22.566 ± 0.03 ^a^	15.806 ± 0.01 ^cd^
20	17.160 ± 0.01 ^g^	14.143 ± 0.02 ^d^	4.113 ± 0.03 ^c^	14.729 ± 0.07 ^d^	16.216 ± 0.05 ^b^
21	17.753 ± 0.02 ^d^	11.326 ± 0.04 ^f^	2.470 ± 0.04 ^e^	11.592 ± 0.02 ^f^	12.302 ± 0.07 ^e^
22	17.410 ± 0.02 ^f^	7.670 ± 0.07 ^h^	1.963 ± 0.04 ^f^	7.917 ± 0.07 ^h^	14.358 ± 0.02 ^d^
23	20.036 ± 0.03 ^a^	21.050 ± 0.03 ^b^	0.340 ± 0.02 ^g^	21.052 ± 0.07 ^b^	0.925 ± 0.02 ^g^
24	17.626 ± 0.03 ^e^	12.426 ± 0.16 ^e^	1.853 ± 0.04 ^f^	12.564 ± 0.05 ^e^	8.481 ± 0.03 ^f^

^1^ MSD (L*) = 0.8357; GA (L*) = 19.831; CV (L*) = 1.49%; MSD (A*) = 1.3119; GA (A*) = 14.445; CV (A*) = 3.21%; MSD (B*) = 1.7905; GA (B*) = 1.422; CV (B*) = 5.10%; MSD (Chroma*) = 1.4006; GA (Chroma*) =14.9373; CV (Chroma*) = 3.32%; MSD (°hue) = 5.9841; GA (°hue) = 14.9373; CV (°hue) = 14.51%. ^2^ MSD (L*) = 0.1634; GA (L*) = 18.662; CV (L*) = 0.31%; MSD (A*) = 0.2553; GA (A*) = 10.144; CV (A*) = 0,89%; MSD (B*) = 0.5103; GA (B*) = 1.6962; CV (B*) = 3.89%; MSD (Chroma*) = 0.2242; GA (Chroma*) = 10.619; CV (Chroma*) = 0.75%; MSD (°hue) = 4.2996; GA (°hue) = 20.7598; CV (°hue) = 7.34%. ^3^ MSD (L*) = 0.0616; GA (L*) = 18.098; CV (L*) = 0.12%; MSD (A*) = 0.3017; GA (A*) = 14.651; CV (A*) = 0.73%; MSD (B*) = 0.1307; GA (B*) = 3.3508; CV (B*) = 1.38%; MSD (Chroma*) = 0.2934; GA (Chroma*) = 15.1239; CV (Chroma*) = 0.69%; MSD (°hue) = 0.6862; GA (°hue) = 13.0493; CV (°hue) = 1.86%. MSD—minimum significant deviation; GA—general average; CV—coefficient of variation; means followed by the same letters do not differ statically by Tukey’s test, at 5% probability.

**Table 3:** Specification coding of red cabbage extracts.

Solvent	Codification	Raw Material	pH Range	Processing Temperature (°C)
Water	01	In natura	4.0	75
	02	70 °C/1 h	4.0	75
	03	In natura	4.0	25
	04	70 °C/1 h	4.0	25
	05	In natura	6.0	75
	06	70 °C/1 h	6.0	75
	07	In natura	6.0	25
	08	70 °C/1 h	6.0	25
Alcohol 25%	09	In natura	4.0	75
	10	70 °C/1 h	4.0	75
	11	In natura	4.0	25
	12	70 °C/1 h	4.0	25
	13	In natura	6.0	75
	14	70 °C/1 h	6.0	75
	15	In natura	6.0	25
	16	70 °C/1 h	6.0	25
Alcohol 70%	17	In natura	4.0	75
	18	70 °C/1 h	4.0	75
	19	In natura	4.0	25
	20	70 °C/1 h	4.0	25
	21	In natura	6.0	75
	22	70 °C/1 h	6.0	75
	23	In natura	6.0	25
	24	70 °C/1 h	6.0	25

## Data Availability

The data presented in this study are available on request from the corresponding authors.

## Sample Availability

Samples of the extracts obtained in this study are available from the authors.
